# The availability and effectiveness of tools supporting shared decision making in metastatic breast cancer care: a review

**DOI:** 10.1186/s12904-018-0330-4

**Published:** 2018-05-11

**Authors:** Inge Spronk, Jako S. Burgers, François G. Schellevis, Liesbeth M. van Vliet, Joke C. Korevaar

**Affiliations:** 1 NIVEL (Netherlands Institute for Health Services Research), P.O. Box 1568, 3500BN Utrecht, The Netherlands; 2 Dutch College of General Practitioners, Utrecht, The Netherlands. School CAPHRI, Department Family Medicine, Maastricht University, Maastricht, The Netherlands; 3 Department of General Practice & Elderly Care Medicine/ Amsterdam Public Health Research Institute, VU University Medical Center, Amsterdam, The Netherlands

**Keywords:** Shared decision making, Metastatic breast cancer, Decision aid

## Abstract

**Background:**

Shared decision-making (SDM) in the management of metastatic breast cancer care is associated with positive patient outcomes. In daily clinical practice, however, SDM is not fully integrated yet. Initiatives to improve the implementation of SDM would be helpful. The aim of this review was to assess the availability and effectiveness of tools supporting SDM in metastatic breast cancer care.

**Methods:**

Literature databases were systematically searched for articles published since 2006 focusing on the development or evaluation of tools to improve information-provision and to support decision-making in metastatic breast cancer care. Internet searches and experts identified additional tools. Data from included tools were extracted and the evaluation of tools was appraised using the GRADE grading system.

**Results:**

The literature search yielded five instruments. In addition, two tools were identified via internet searches and consultation of experts. Four tools were specifically developed for supporting SDM in metastatic breast cancer, the other three tools focused on metastatic cancer in general. Tools were mainly applicable across the care process, and usable for decisions on supportive care with or without chemotherapy. All tools were designed for patients to be used before a consultation with the physician. Effects on patient outcomes were generally weakly positive although most tools were not studied in well-designed studies.

**Conclusions:**

Despite its recognized importance, only two tools were positively evaluated on effectiveness and are available to support patients with metastatic breast cancer in SDM. These tools show promising results in pilot studies and focus on different aspects of care. However, their effectiveness should be confirmed in well-designed studies before implementation in clinical practice. Innovation and development of SDM tools targeting clinicians as well as patients during a clinical encounter is recommended.

## Background

Breast cancer is the most commonly diagnosed cancer type among women worldwide and the fifth cause of cancer related deaths [1]. In metastatic breast cancer care many complex decisions need to be made, of which most are preference-sensitive [2, 3]. Important treatment decisions include for example whether or not to start chemotherapy or targeted therapy [4].

Shared decision making (SDM) is an approach in which health care providers and patients share the best evidence when facing decisions, and patients are encouraged to be actively involved in decision making [5, 6]. SDM has been identified as an important element for good advanced cancer care [7]. Most cancer patients prefer to participate in decision making [8, 9]. Among patients with advanced cancer, women with breast cancer in particular wish to be actively involved in decision making [10]. SDM is associated with positive patient outcomes, including knowledge regarding available options, perceived quality of care [11, 12], and quality of life [13].

The use of tools might support active participation of patients in decision-making. Examples of such instruments are 1) decision aids (DAs) which are designed to be used by patients before doctor visits to prepare for decision making [14–16], and 2) tools to be used by both health care providers and patients during clinical encounters [17, 18]. DAs are developed to support patients in decision making by providing an overview of the available (treatment) options and their associated outcomes [15, 19]. There are many types of DAs, such as video or audiotapes, patient letters, computer programs, leaflets, and interactive media [12].

The tools designed to be used during consultation with a health care provider have been developed to facilitate a conversation between health care providers and patients about the relevant (treatment) options. In general, these tools are brief and present a summary of available options. Examples are decision boards, bar charts, option grids and consult decision aids [17, 18, 20–22].

The aims of this study were 1) to make an inventory of instruments and tools, including DAs and tools used during clinical encounters, that are currently available for supporting SDM in metastatic breast cancer care and 2) to evaluate the effectiveness of these tools based on published studies.

## Methods

Three strategies were used to identify tools for supporting SDM in metastatic breast cancer. First, a systematic search of relevant databases was undertaken, secondly an internet search was conducted and lastly experts who appeared in the searches were contacted.

### Systematic search

#### Search strategy

A systematic literature search was conducted in Cinahl, Medline, PsychInfo and Pubmed to identify relevant articles published between 1 January 2006 and 18 January 2017. This time frame was chosen as we were looking for tools that are still clinical relevant and up-to-date. If there were instruments developed before 2006 that are still relevant, we would have find them in either later publications, via our internet search, or via the experts that we have approached. The search strategy was developed in collaboration with an experienced librarian and checked by an expert in the field. It combined terms covering the areas of breast cancer (breast cancer; breast carcinoma; breast neoplasms), advanced cancer (advanced cancer, metastatic cancer, palliative care), decision making (decision making, decision support, decision aid, shared decision) (Appendix 1). Hand-searching of reference lists of included articles was conducted to identify additional studies.

#### Study selection

The search was performed by one reviewer (IS), and after removal of duplicates, irrelevant articles were eliminated on the basis of title and abstract. Ten percent was independently evaluated by two reviewers (IS and JK). There was no disagreement between the reviewers on inclusion. Therefore, the remaining abstracts were evaluated by one reviewer (IS). Screening of full text of relevant articles was independently performed by two reviewers (IS and JK). Disagreements were resolved by discussion with a third reviewer (FS).

#### Inclusion criteria

Research articles and (systematic) reviews on studies conducted in advanced breast cancer patients, written in any language and published in a peer-reviewed journal were included for review. Studies needed to focus on the development and/or evaluation of an initiative or tool that focused on i) information provision about decisions, ii) decision making process, or iii) eliciting treatment preferences in metastatic breast cancer care. Outcomes included in the studies had to be any i) patient-reported outcome, or ii) health outcome.

#### Data extraction

Characteristics of tools (name, country, description, target population, type of tool, decision on which tool focusses), study characteristics (first author, year of publication, study size, patients characteristics, study design, outcome measures) and patient-reported and health outcomes were independently extracted by two reviewers (IS and JK).

#### Study quality

Quality of the studies evaluating the tools was independently assessed by two reviewers (IS and JK) using the Grading of Recommendations Assessment, Development and Evaluation (GRADE) methodology [23]. This methodology classifies evidence into four levels of quality (high to very low). First the studies were classified based on their design, with high quality for randomized control trials and low quality for observational studies. These initial grades can be downgraded or upgraded after assessment of their weaknesses and strengths. The five downgrading criteria are risk of bias, indirectness of evidence, inconsistency of results imprecision of results, and publication bias. The three upgrading criteria are large magnitude of effect, dose-response, and opposing residual confounding or bias. Based on the up- and downgrading criteria, the final evidence grade was determined [23].

### Internet search and consultation of experts

An internet search was performed and experts were approached to complement the systematic literature search using the same inclusion criteria. Google searches covering the areas metastatic breast cancer (advanced breast cancer, metastatic breast cancer, palliative breast cancer care) and decision making (decision making, decision support, decision aid, shared decision) were carried out and websites presenting an overview of decision aids were studied (http://www.med-decs.org/, https://decisionaid.ohri.ca/). National and international experts who appeared in the systematic literature and internet searches were approached via email and asked whether they were aware of tools, instruments or initiatives supporting SDM in metastatic breast cancer care. From the tools identified by internet searches and experts the same characteristics were extracted as from those identified by the systematic literature search.

## Results

**Fig. 1:**
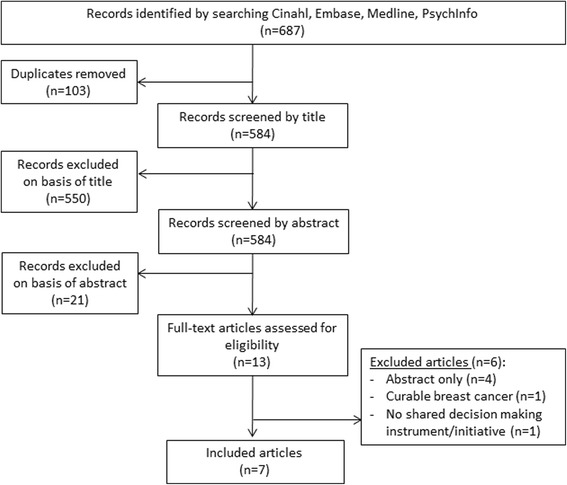
Flowchart outlining article selection process

The initial literature search resulted in 687 potentially relevant articles. After removal of duplicates and elimination of non-eligible papers based on title and abstract, 13 full-text articles were considered, of which seven were included for review (Fig. 1). The seven articles described five different tools. In addition, the internet search revealed two relevant tools. All 17 experts approached responded and identified one additional relevant tool (Table 1).

**Table 1:** Overview of tools for shared decision making in metastatic breast cancer

Name tool/short description	Country	Source^a^	Specific for metastatic breast cancer	Description of tool
CONNECT	USA	S	No	A communication aid that assesses patient preferences and values, and includes communication skills training, plus summary report to the physician.
Decision aid on first, second, third and fourth line chemotherapy	USA	S	No	State-of-the-art tables with information for patients with advanced breast, lung, colon, and hormone-refractory prostate cancers
Decision aid on first-line chemotherapy	Australia and Canada	E, S	Yes	A DA presenting options of supportive care, with or without chemotherapy. Potential benefits and side effects of different chemotherapy regimens, and evidence-based prognostic estimates are described, and a values clarification exercise is included.
Decision aid on second-line chemotherapy	The Netherlands	E, I, S	Yes	A DA describing the adverse events, response of the cancer and survival of supportive care with or without second-line palliative chemotherapy.
Decision aid ‘metastatic breast cancer’	The Netherlands	I	Yes	A booklet presenting information on therapies and supportive treatment in metastatic breast cancer. It provides information on what characteristics define how metastatic breast cancer can be treated and shows other patients arguments for and against treatment.
Consultation guide	The Netherlands	E	No	A booklet with sample questions and an instrument for value clarification.
Living with Metastatic Breast Cancer: Making the Journey Your Own.	USA	S	Yes	A thirty-minute video/DVD and accompanying booklet depicting the experiences of 4 women living with metastatic breast cancer.

*Note.* ^a^Source*: S* systematic search, *E* experts, *I* internet search

*DA* decision aid

In total, seven tools were identified (Table 1). Three were developed in the USA, three in the Netherlands and one in Canada and Australia. Four tools were specifically designed for metastatic breast cancer, the others for metastatic cancer patients in general. Three tools focused on the decision on whether or not to start chemotherapy [24–27]. The other four focused on all possible decisions during the entire metastatic breast cancer care trajectory. All tools were developed for patients to be used before consultation with their health care provider. Only one tool [28] provided a summary report to the health care provider which could be discussed during a consultation.

The content of five out of seven tools was evaluated in published studies (Table 2). Of four of these, the effectiveness was studied as well. CONNECT, the communication aid from Meropol et al. was tested in a randomized clinical trial [28]. Outcome measures included consultation content, treatment outcome expectations, decisional conflict, patient satisfaction with the content and format of the communication, and satisfaction with the survey and/or communication skills training. CONNECT made it easier for patients to make treatment decisions (*P* = 0.003) and patients were more satisfied with their decision (*P* < 0.001), with physician communication (*P* = 0.026), with discussion regarding support services (*P* = 0.029) and quality of life concerns (*P* = 0.042), but not with discussion of diagnosis/prognosis, treatment options, or support/community services. The DA of Oostendorp et al., [25, 26, 29] was evaluated in a randomized clinical trial. Primary outcome measures included several measurements on patient’s well-being on which the DA had no statistically significant effect. The DA was associated with stronger treatments preferences of patients (*P* = 0.030) and with increased subjective knowledge (*P* = 0.022), but not with any of the other secondary outcomes measures. The two other tools [27, 30] were tested in pilot studies without control groups. The DA of Smith et al. [27] assessed whether patients choose to use the DA, investigated the knowledge of patients about the disease and treatment and examined whether the information of the DA was helpful and if the patient wanted to share the information with the physician. All except one patient used the DA and knowledge about the cure of advanced cancer improved after using the DA (*P* = 0.15). Most patients found the information helpful and almost all patients wanted to share information with their physician after use of these DAs, which might result in SDM. The study on the DA developed by Sepucha et al. [30] evaluated acceptability of the DA and its impact on decisions. The DA was rated acceptable, did not increase distress (*P* = 0.34) and the treatment goal was most often to lengthen life. Most patients (88%) wanted to be involved in shared decision making, however, only 41% found that decision making was shared and 38% achieved their desired level of participation in decision making. The content of the tool and attitudes towards the tool developed by Chiew et al. was evaluated by both patients and medical oncologists [24]. The patients concluded that the DA was acceptable and helpful and the majority recommend the use of this DA to others. Also the oncologists were positive about the DA and found the DA appropriate for all or most patients.

**Table 2:** Evaluated tools

Name tool/short description	First author (year)	Study population^a^	Design	Decision aid outcome measures	Outcome	GRADE
CONNECT	Meropol (2013)	Metastatic cancer patients, *n* = 629 (F:48%), mean age: 59 year	Randomized clinical trial with 3 arms	Consultation content, treatment outcome expectations, decisional conflict, patient satisfaction with the content and format of the communication, and satisfaction with the survey and/or communication skills training	• Treatment decisions were easier to reach (*P* = 0.003) • Patients were more satisfied with decisions (*P* < 0.001) • Patients were more satisfied with physician communication (*P* = 0.026) • Patients were more satisfied with discussion regarding support services (*P* = 0.029) and quality of life concerns (*P* = 0.042) • No statistically significant differences in satisfaction regarding discussion of diagnosis/prognosis, treatment options, or support/community services.	Low
Decision aid on first, second, third and fourth line chemotherapy	Smith (2011)	Patients with metastatic breast, colorectal or lung cancer, *n* = 27 (F:56%), mean age: 63 year	Pilot pretest, posttest study	Number of patients who opt for full disclosure once they viewed the DA The amount of information patients have about cure, response rates, and symptom control; the impact of truthful information on hope, whether the information was deemed helpful to the patient; and whether the patient want to share the information with a physician	• 96% of the patients chose to complete the DA • The proportion of patients who thought that advanced cancer could be cured reduced from 52 to 31% (*P* = 0.15) • 87% of the patients overestimated the effect of palliative chemotherapy • No distress was noted and hope did not change • 74% found the information helpful • 93% wanted to share the information with their family and physician	Very low
Decision aid on first-line chemotherapy	Chiew (2008)	Metastatic breast cancer patients, *n* = 17 (F:100%), median age: 58 year Medical oncologists, *n* = 7	Pilot observational study	Patients’ attitudes toward the DA, and oncologist feedback regarding attitudes toward the DA.	• The DA was rated acceptable and helpful. • The DA contains an appropriate amount of information, and the length is appropriate • 94% of the patients would recommend use of the DA to others • Oncologists received the DA positively and found it appropriate for all or most patients	Very low
Decision aid on second-line chemotherapy	Oostendorp (2017)	Patients with metastatic breast or colorectal cancer, *n* = 128 (F:63%), median age: 62 year	Randomized clinical trial	Anxiety, depression, general health, cancer worries, health-related quality of life, coping styles, amount of information received, satisfaction with quality of information, subjective knowledge, treatment preference, decision satisfaction and uncertainty, decision control and treatment attitudes.	• The DA had no adverse impact on patient’s well-being • Use of the DA was associated with stronger treatment preferences (*P* = 0.030) and increased subjective knowledge (*P* = 0.022) • No statistically significant differences in anxiety, depression, general health, cancer worries, health-related quality of life, coping styles, amount of information received, satisfaction with quality of information, decision satisfaction and uncertainty, decision control and treatment attitudes.	Moderate
Living with Metastatic Breast Cancer: Making the Journey Your Own	Sepucha (2009)	Metastatic breast cancer patients, *n* = 32 (F:100%), median age: 55 year	Pilot pretest, posttest study	Use and acceptability of DA, distress, treatment goals, and preference for and actual participation in decision	• The DA was rated acceptable and did not increase distress (*P* = 0.34) • Most patients (88%) desired to share decision making with their physician • 41% of the patients found that decision making was shared • 38% achieved their desired level of participation • The main goal of treatment was most often to lengthen life	Very low

*Note.* ^a^Study population: *n* sample size, *F* female, *NA* not applicable

*DA* decision aid

The quality of five evaluation studies could be assessed. According to the GRADE approach, the quality of the studies ranged between moderate and very low (Table 2). All studies had noteworthy shortcomings, mainly because of the study design. Two had a randomized design and the others were observational studies [24, 27, 30]. The quality of the three observational studies was downgraded to ‘very low’ due to small samples sizes, unclear descriptions of inclusion criteria and lack of information on loss to follow-up. The quality of the studies with a randomised design was downgraded as well [28, 29]. Both studies had a high drop-out rate. And one defined no primary outcome and presented selective results as two of the intervention arms were combined to obtain significant results.

## Discussion

This review identified seven tools to support SDM in metastatic breast cancer care. All were designed to be used independently by patients before consulting a physician. None was developed to be used by both a health care provider and patient during a clinical encounter, although one tool provided a summary report for the physician which could be discussed during a consultation. In general, the identified tools had positive effects on patient satisfaction with their treatment decision and on patients’ desire to share information with their physician. However, it is unclear whether they encourage SDM during a clinical encounter as this was not studied. The effectiveness of the included tools was barely studied. Evidence from the included studies was in general low due to multiple sources of bias, which may have skewed the results.

The revealed tools to support patients in SDM have some limitations. The effectiveness of only four of them was evaluated [27–30]. Of these, the one with the highest level of evidence was not effective [29]. The other tool with a somewhat higher level of evidence of effectiveness is not available anymore [28] as the tool was not kept up-to-date. The two remaining tools might be useful in clinical practice as their results are promising in pilot studies. These tools could be used next to each other as the DA of Smith et al. [27] focuses on chemotherapy, whereas the DA of Sepucha et al. [30] shows the experiences of four women living with metastatic breast cancer. A limitation of these tools is that they were only tested in a pilot study without a control group. Further testing of these tools in better designed studies is required before they are implemented. The consultation guide presenting information on therapies and supportive treatment in metastatic breast cancer, was not evaluated, but might also be useful for patients with metastatic breast cancer.

Despite the calls for integrating SDM in clinical practice, implementation of SDM into daily care is lacking [31–34]. The lack of available SDM supporting tools and time concerns might be barriers for implementation [6]. Our review shows the availability of a few tools to be used by patients before visiting the physician and the lack of tools to be used during a clinical encounter in metastatic breast cancer care. In general, tools to be used by patients before visiting the health care provider lead to better understanding of choices, however, yet are not enough to guarantee SDM [14, 35]. In order to facilitate SDM during a clinical encounter, SDM tools for both health care providers and patients have been designed [35–37]. For curative breast cancer and other tumour types, such tools are available [17, 18]. These tools make options more visible, enhances patients confidence and involvement, and clinicians find it easier to implement SDM in practice [18]. For decision making in metastatic breast cancer care, there is a pressing need for similar tools as many complex decisions have to be made and alignment of care with patient preferences is necessary.

When developing, testing and implementing tools for SDM during a clinical encounter, several recommendations can be made. First, tools should be based on the best available scientific evidence and being kept up-to-date [25, 38–40]. Second, patients should be included in their development to ensure the tools are user-friendly and understandable [41]. Third, the impact on patient outcomes should be evaluated. Fourth, the conditions for appropriate use of tools in clinical practice should be realized, e.g. clinical teams should recognise the importance of SDM and should be trained in SDM [6, 42], and sufficient time should be available to use a tools for SDM during a clinical encounter [6, 43–45].

## Conclusions

Only two tools for SDM in metastatic breast cancer care were positively evaluated on effectiveness and are currently available. These are developed to be used by patients before consulting the physician. None have been tested in well-designed studies. These tools show promising results in pilot studies and focus on different aspects of care. However, their effectiveness should be confirmed in well-designed studies before implementation in clinical practice. Innovation and development of SDM tools targeting clinicians as well as patients during a clinical encounter is recommended.

## Appendix 1. Search strategy

**Table 3:** Search in Pubmed (date: 18 January 2017)

Search strategy		Number of hits
	Breast cancer	
#1	“breast cancer” [tiab]	
#2	breast neoplasms [mesh]	
#3	“breast neoplasm*” [tiab]	
#4	“breast carcinoma” [tiab]	
#5	“breast tumor” [tiab]	
#6	“breast tumour” [tiab]	
#9	#1 OR #2 OR #3 OR #4 OR #5 OR #6 OR #7 OR #8	307,107
	Palliative care	
#10	palliative care [mesh]	
#11	palliative [tiab]	
#12	Hospice Care [mesh]	
#13	hospice [tiab]	
#14	end-of-life [tiab]	
#15	terminal [tiab]	
#16	incurable [tiab]	
#17	Terminal Care [mesh]	
#18	“early palliative care” [tiab]	
#19	“serious illness” [tiab]	
#20	“advanced cancer” [tiab]	
#21	“metastatic cancer” [tiab]	
#22	metastasis [tiab]	
#23	Neoplasm Metastasis [MeSH Terms]	
#24	#10 OR #11 OR #12 OR #13 OR #14 OR #15 OR #16 OR #17	770,461
	Decision making	
#19	“decision making”[tiab]	
#20	“decision support”[tiab]	
#21	“decision aid*”[tiab]	
#22	“choice behavior”[tiab]	
#23	“choice behaviour”[tiab]	
#24	(((((shared)[tiab] OR sharing)[tiab] OR informed[tiab]))) AND ((decision*[tiab]) OR choice*[tiab])	
#25	#19 OR #20 OR #21 OR #22 OR #23 OR #24	116,279
#26	#9 AND #18 AND #25	314
	limit #26 to (humans and yr. = “2006–2017”)	181

*Note*. This initial search strategy was adapted to Cinahl, Medline and PsychInfo

## Abbreviations

DA: Decision aid
GRADE: Grading of Recommendations Assessment, Development and Evaluation
SDM: Shared decision-making
